# Ultrasonic surgical aspiration (CUSA®) for laparoscopic excision of endometriosis: a prospective case series demonstrating safety and precision in fertility-preserving surgery

**DOI:** 10.3389/fsurg.2025.1735940

**Published:** 2025-12-16

**Authors:** Márcia Vieira-Coimbra, Hélder Ferreira

**Affiliations:** 1 Serviço de Ginecologia e Obstetrícia, Unidade Local de Saúde (ULS) Viseu Dão-Lafões, Viseu, Portugal; 2 Hospital da Luz Arrábida, Porto, Portugal; 3 Serviço de Ginecologia, Departamento da Mulher e da Medicina Reprodutiva, Centro Materno Infantil do Norte (CMIN), ULS Santo António, Porto, Portugal; 4 School of Medicine and Biomedical Sciences (ICBAS), University of Porto, Porto, Portugal

**Keywords:** endometriosis, laparoscopic surgery, cavitron ultrasonic surgical aspirator (CUSA), fertility preservation, energy-based surgical devices, thermal injury, minimally invasive gynecology

## Abstract

**Introduction:**

Endometriosis affects ∼10% of women of reproductive age, often causing chronic pelvic pain and infertility. Conventional energy devices risk thermal injury and bleeding, particularly in fertility-preserving surgeries. The Cavitron Ultrasonic Surgical Aspirator (CUSA®) selectively fragments tissue with minimal thermal spread. This prospective case series evaluates CUSA's safety and effectiveness in endometriosis surgery.

**Material and methods:**

Fifteen women with suspected peritoneal, deep-infiltrating, or diaphragmatic endometriosis underwent laparoscopic excision exclusively using CUSA at a single center (Jan 2024–Jan 2025). Outcomes included operative time, blood loss, pain score change, recovery time, and complications.

**Results:**

Mean CUSA time was 8.5 ± 3.0 min with a median blood loss was less than10 mL. No intraoperative complications or conversions occurred. Surgeon-reported performance scores demonstrated high procedural efficiency and manageable technical challenges. At the first follow-up visit (6–8 weeks postoperatively), Numeric Pain Rating Score decreased by 3.2 points from 6.9 ± 1.4 to 3.7 ± 1.0, indicating marked symptom relief. All patients resumed daily activities within 3 days. In all patients, all visible lesions suggestive of endometriosis were excised intraoperatively, and histology confirmed endometriosis in all cases. Discussion: CUSA allows precise and safe laparoscopic excision of endometriosis with minimal bleeding, absence of perioperative complications, and significant short-term pain reduction. These findings demonstrate the feasibility and short-term safety of the technique and support its potential value in fertility-preserving surgery. However, given the small sample size, single-center design, and limited follow-up, the results should be interpreted with caution. Future multicenter studies with larger cohorts and reproductive outcome assessment are needed to confirm these preliminary findings.

## 1 Introduction

Endometriosis is a chronic, estrogen-dependent condition affecting approximately 10% of reproductive-age women (1). It often causes pelvic pain, infertility, and reduced quality of life (2). When medical management fails, surgical excision is the preferred treatment. In such cases, particularly when fertility preservation is a concern, the procedure must be performed with high precision to avoid damage to delicate reproductive structures (1). In this context, the choice of surgical energy source plays a pivotal role in balancing efficacy and safety.

Energy-based excision techniques are central to gynecologic surgery, providing effective tissue dissection and hemostasis (3). However, conventional modalities such as monopolar and bipolar electrosurgery are limited by thermal spread, which can cause unintended injury to surrounding tissues—a particular concern when operating near delicate reproductive structures (4, 5). This risk is amplified in fertility-preserving procedures, where precise excision is critical. Even advanced high-frequency bipolar sealing devices can produce considerable thermal spread, increasing the risk of collateral damage (6). These drawbacks highlight the need for newer technologies that enable more precise tissue to target minimal thermal damage, particularly in complex gynecologic surgeries.

**Figure 1:**
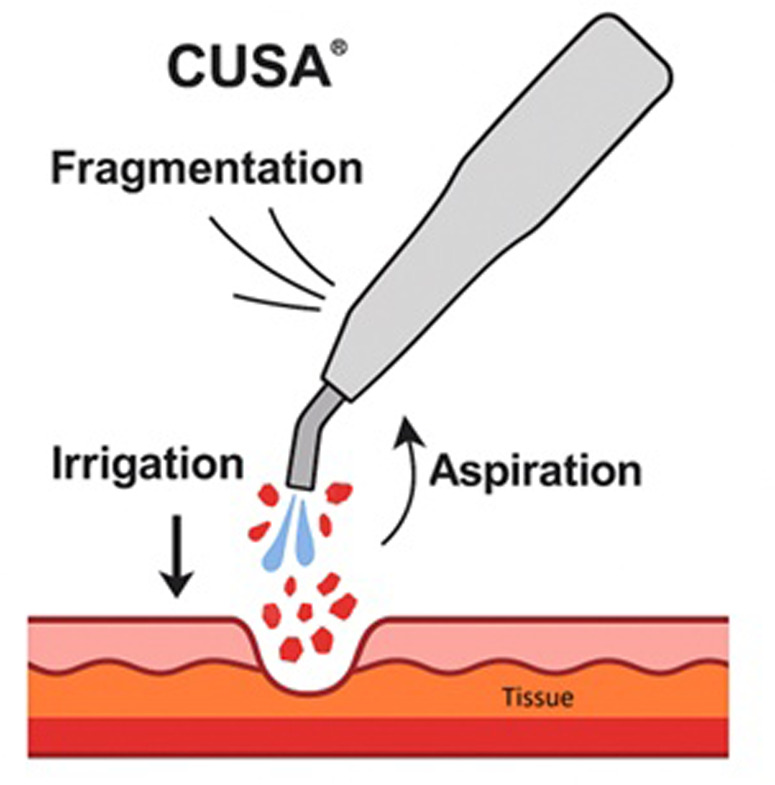
Schematic representation of the cavitron ultrasonic surgical aspirator (CUSA®) mechanism of action, showing fragmentation of hydrated tissue by ultrasonic vibration, simultaneous irrigation to flush debris, and aspiration to maintain a clear surgical.

The Cavitron Ultrasonic Surgical Aspirator (CUSA®) is an advanced surgical device that uses ultrasonic vibration (23–36 kHz) to selectively fragment hydrated tissue (7). It is based on three main mechanisms principles: fragmentation, irrigation, and aspiration (Figure 1). It utilizes ultrasonic waves and vibrations to induce cavitation, breaking hydrogen bonds and leading to the denaturation of tissue proteins (8, 9). Simultaneously, irrigation flushes the fragmented material, while aspiration clears debris, maintaining visibility and efficiency during surgery (8).

One of CUSA's main advantages is its ability to selectively fragment tissues with high water content and weak cellular cohesion, while preserving collagen-rich structures such as vessels, nerves, and ducts (7, 8). This selectivity is particularly valuable in complex procedures involving deep or fibrotic lesions near vital structures, where surgical precision is essential (10). Additionally, CUSA offers limited thermal spread, reduced intraoperative bleeding, and better visualization of the surgical field (7, 10, 11).

While widely used in hepatobiliary, neurosurgical and cardiothoracic procedures, its use in gynecology—particularly in endometrioses—remains relatively underreported. Some studies have reported its application in selected gynecologic surgeries, such as diaphragmatic stripping during advanced ovarian-cancer debulking (12), palliative debulking of recurrent vaginal malignancies (13), laparoscopic nerve-sparing radical hysterectomy (14), and procedures aimed at preserving postoperative vesicourethral function (15). More recently, its use has also been described in the laparoscopic excision of deep-infiltrating (16) and diaphragmatic endometriosis (17), where precision and tissue preservation are particularly critical.

This prospective study evaluates the safety, precision, and clinical effectiveness of CUSA for laparoscopic excision of endometriosis, focusing on minimal blood loss, preservation of surrounding structures, and postoperative recovery.

## 2 Material and methods

### 2.1 Study design

This was a prospective, single-center descriptive case series conducted at Hospital da Luz Arrábida, Porto, Portugal, between January 2024 and January 2025. A total of 15 women were consecutively enrolled. Inclusion criteria were: (i) age 18–50 years and (ii) clinical and imaging (transvaginal ultrasound ± MRI) suspicion of peritoneal, deep-infiltrating, or diaphragmatic endometriosis suitable for laparoscopic excision with CUSA. Exclusion criteria included prior pelvic malignancy, contraindication to laparoscopy, current pregnancy, refusal of informed consent, or cases requiring full-thickness bowel or urinary tract resection (segmental bowel resection, discoid excision, ureterolysis, or partial cystectomy). All enrolled patients underwent laparoscopic excision with CUSA and were included in the final analysis.

### 2.2 Surgical procedure

All surgeries were performed by a single experienced surgeon, who had previously completed 10 procedures using the CUSA® device. The CUSA Excel+® ultrasonic aspirator (Integra LifeSciences®, Princeton, NJ, USA) was used exclusively for lesion excision and adhesiolysis—no monopolar, bipolar, or laser devices were employed. An initial pelvic inspection was performed through a four-port laparoscopic approach, followed by intraoperative staging according to the Enzian classification. Visible endometriotic lesions and adhesions were then treated using a 10-mm, 23 kHz CUSA Excel + probe with continuous irrigation and aspiration. Lesion sites included peritoneum, uterosacral ligaments, rectovaginal septum, torus uterinus, ovaries (infiltrating ovarian endometriosis), and diaphragm. All surgical specimens were routinely submitted for histopathological analysis to confirm the diagnosis and evaluate histological features.

### 2.3 Data collection

For each case, we prospectively recorded skin-to-skin operative time, estimated blood loss, and surgical performance scores (1 = very easy/highly effective to 5 = very difficult/ineffective) for ease of lesion ablation, ease of adhesiolysis, and overall effectiveness of CUSA, as assessed by the lead surgeon. Ease-of-ablation was assessed based on tissue fragmentation, bleeding control, and time/effort required; ease-of-adhesiolysis on adhesion characteristics, separation from critical structures, and instrument maneuverability; and overall effectiveness on lesion removal completeness, preservation of vital structures, hemostasis, and surgical efficiency. The Numerical Rating Scale (NRS) was applied preoperatively to evaluate pain levels.

Postoperative data included complications (Clavien–Dindo classification), hospital stay, and any reoperation within 30 days. At the 4–6 week follow-up, NRS pain score was reassessed, along with time to resume daily and professional activities.

So primary outcomes were CUSA dissection time, blood loss, ease-of-ablation score, ease-of-adhesiolysis score, and overall effectiveness score. Secondary outcomes included hospital stay, time to resume daily/professional activities, change in NRS pain score, and postoperative complications.

### 2.4 Data analysis

Data were analyzed descriptively, as appropriate for the small sample size and exploratory design of this case series. No hypothesis testing or power analysis was performed. Continuous variables are reported as median (interquartile range) and categorical variables as frequencies and percentages. Tabulations were generated in Microsoft Excel 365®, and graphs in SPSS® version 29.

### 2.5 Ethical considerations

The study protocol was approved by the Local Ethics Committee of Hospital da Luz Arrábida. All procedures were conducted in accordance with the Declaration of Helsinki and national data protection regulations.

## 3 Results

### 3.1 Sociodemographic and baseline characteristics

Fifteen patients meeting inclusion criteria underwent laparoscopic excision using CUSA. Sociodemographic and baseline characteristics of the study cohort are summarized in Table 1. The study cohort comprised 15 women aged 21–39 years (mean: 29y ± 5). The most commonly reported symptoms were dyspareunia (*n* = 15) and dysmenorrhea (*n* = 14), followed by dyschezia (*n* = 8) and scapulodynia (*n* = 4). The mean preoperative NRS score was 6.9 ± 1.4, indicating moderate to severe pain levels at baseline.

**Table 1:** Sociodemographic and baseline characteristics of our participants.

Characteristic	Value
Participants, *n*	15
Age (Mean ± SD)	29 ± 5 (range 21–39)
Presenting symptoms, *n* (%)	
• Dysmenorrhea • Dyspareunia • Dyschezia • Scapulodynia	14 (93%) 15 (100%) 8 (53%) 2 (13%)
Preoperative NRS Pain Score (Mean ± SD)	6.9 ± 1.4

NSR, numeric rating scale; SD, standard deviation.

### 3.2 Intra-operative findings

The CUSA phase of the procedure had a mean duration of 8.5 ± 3.0 min (range 4–14 min), with a median estimated blood loss of less than 10 mL (no case exceeded 20 mL). The mean ease-of-ablation score was 1.3 ± 0.5, the ease-of-adhesiolysis score of 1.7 ± 0.7, and overall effectiveness score of 1.4 ± 0.6, indicating high procedural efficiency and manageable technical challenges. No intraoperative complications or conversions occurred. In all patients, all visible lesions suggestive of endometriosis, including both superficial and deep lesions, were excised using CUSA. The detailed results are presented in Table 2. The location and extent of endometriotic lesions were documented using the Enzian classification (Supplementary Table 1).

**Table 2:** Intraoperative parameters collected for each patient in CUSA-assisted laparoscopic excision of endometriosis.

Parameter	Value
CUSA time (min)	8.5 ± 3.0 (range 4–14)
Estimated blood loss (mL)	<10 median (max 20)
Ease of ablation score	1.3 ± 0.5 (range 1–2)
Ease of adhesiolysis score	1.7 ± 0.7 (range 1–3)
Overall effectiveness score	1.4 ± 0.6 (range 1–3)

Blood loss is estimated suction volume minus irrigation; “<10 mL” indicates negligible bleeding. Surgeon ratings are on a 1–5 scale (1 = very easy/highly effective; 5 = very difficult/ineffective).

**Figure 2:**
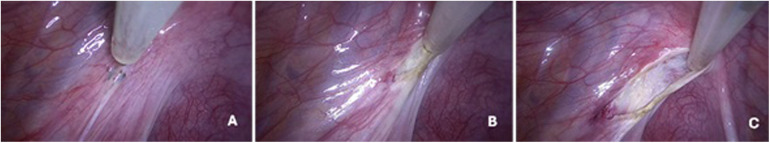
Representative intraoperative images of CUSA from our series. **(A)** Pre-procedural view before the start of lesion excision. **(B,C)** Intraoperative views demonstrating tissue fragmentation, irrigation, and aspiration during the use of CUSA.

Figure 2 shows a representative intraoperative image of the CUSA from our series.

### 3.3 Post-operative course

**Figure 3:**
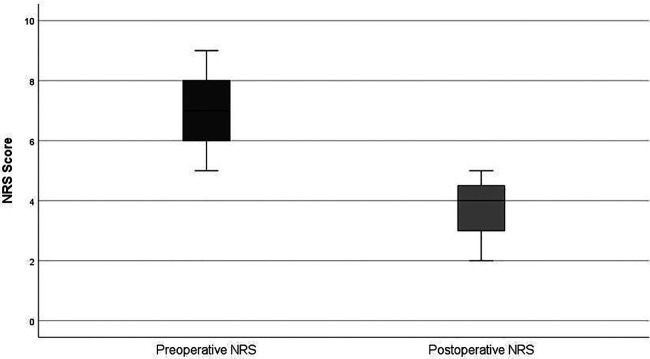
Comparison of preoperative and postoperative pain scores (NRS).

All patients were discharged after one night (median length of stay = 1 day). Daily activities were resumed within 2–3 days and return to professional activities occurred after a mean of 7 days. No early (≤30 days) or late (>30 days) postoperative complications or reoperations were observed. At the first follow-up visit (6–8 weeks postoperatively), Numeric Pain Rating Score decreased by 3.2 points (46%), from 6.9 ± 1.4 to 3.7 ± 1.0, indicating marked symptom relief (Figure 3). Histopathological analysis confirmed endometriosis in all surgical specimens. Fertility outcomes were not assessed due to the limited follow-up period.

## 4 Discussion

Our findings indicate that laparoscopic excision of endometriosis using CUSA is a safe and efficient alternative to conventional energy devices, particularly in fertility-preserving surgery. Blood loss was minimal (median <10 mL), operative time acceptable (mean 8.5 min) and no perioperative complications or reinterventions occurred. Postoperatively, symptoms improved significantly, with a mean pain reduction of 3.2 points (46%), supporting its role in minimizing bleeding while maintaining procedural efficiency and achieving symptom relief. To our knowledge, this is the first prospective series focusing exclusively on fertility-preserving laparoscopic CUSA excision for endometriosis, with no conversions and exceptionally low blood loss, distinguishing it from previous reports.

As a prospective descriptive case series without a control group, this study was not intended to provide comparative efficacy data but rather to document the feasibility, safety, and early outcomes of the CUSA® technique in fertility-preserving endometriosis surgery. While limited in scale, this work contributes preliminary evidence supporting the technical applicability and tissue-sparing potential of ultrasonic aspiration in this setting.

Our results, align with previous reports, demonstrate the efficacy of CUSA in gynecological surgery. Vasquez et al. reported effective lesion ablation with blood loss below 50 mL in 15 laparoscopic cases treated with a first-generation CUSA, with operative times comparable to our findings (16). Further reviews have highlighted similar advantages in complex adhesiolysis and cytoreductive surgery for ovarian or vaginal recurrences (12, 13). More recently, Hao et al. demonstrated that integrating CUSA into laparoscopic nerve-sparing radical hysterectomy resulted in reduced blood loss, shorter catheterization time, and decreased hospital stay, without prolonging surgery duration (14). These consistent outcomes reinforce the utility of CUSA in managing complex gynecological cases.

The use of CUSA in endometriosis surgery provides several advantages, particularly in patients desiring fertility preservation. The device's ability to fragment hydrated endometriotic tissue through cavitation while sparing collagen-rich vessels and nerves reduces bleeding and minimizes the need for additional coagulation (7). Additionally, the continuous irrigation–aspiration system maintains a clean surgical field and dissipates heat (7), which may explain the absence of thermal injuries observed in our cohort. Ultra-low blood loss and the lack of thermal spread are particularly important for preserving ovarian reserve and tubal function, which are crucial for fertility. In our series, the median blood loss was <10 mL, which is notably lower than that reported with conventional energy devices, where mean blood loss can reach 88.5 mL (range 82–92 mL) (18), highlighting CUSA's potential to minimize intraoperative bleeding. Moreover, the shorter operative duration translates into reduced anesthetic exposure and potentially lower procedural costs.

The potential role of CUSA in ovarian endometrioma surgery also warrants consideration. Traditional cystectomy, widely used for the management of ovarian endometriomas, may inadvertently remove normal ovarian cortex and expose the ovary to bleeding, local hypoxia, and thermal injury from hemostatic energy devices, all of which can negatively affect ovarian function (19). In our cohort, however, ovarian involvement corresponded to infiltrating ovarian surface foci rather than true endometriomas. Nevertheless, the selective tissue fragmentation and minimal thermal spread characteristic of CUSA make its potential application in endometrioma surgery theoretically plausible and potentially advantageous for preserving ovarian reserve. Existing evidence is limited to early reports describing the ablation of endometriomas with CUSA (16), but these observations highlight an important direction for future research, particularly in fertility-preserving settings.

Despite these advantages, it is important to acknowledge that CUSA is not entirely free from potential risks. Although ultrasonic aspiration is designed to minimize bleeding and collateral tissue damage through highly localized energy delivery, a residual risk of hemorrhage or unintended injury to adjacent structures persists, even if substantially reduced. Biomechanical simulation studies demonstrate that ultrasonic energy can generate limited thermal and vibratory effects in surrounding tissues, underscoring the need for careful power modulation and controlled application (20). Likewise, clinical data indicate that minor bleeding may still occur during ultrasonic aspiration, confirming that these risks, although low, cannot be completely eliminated (21).

Compared to conventional energy devices, such as monopolar and bipolar electrosurgery, CUSA offers a significant advantage by minimizing thermal damage. Conventional techniques, while effective for tissue excision and hemostasis, are associated with the risk of collateral thermal injury to surrounding tissues, which is particularly important in fertility-preserving surgeries (6). In contrast, CUSA's ultrasonic cavitation mechanism enables targeted tissue removal with minimal heat propagation, thereby reducing the risk of damaging adjacent anatomical structures (7, 8). Compared to CO₂ laser, CUSA is more widely available and cost-effective while offering similar precision and minimal thermal spread (7, 22). Among other advanced energy systems, plasma-based energy devices also minimize injury to adjacent structures. However, their use is associated with higher healthcare costs, mainly due to expensive consumables and maintenance requirements, which can limit their cost-effectiveness, particularly in resource-constrained settings (23). Advanced bipolar systems (e.g., LigaSure™, EnSeal™) also represent widely adopted alternatives in gynecologic laparoscopy, allowing reliable vessel sealing up to 7 mm and shortening operative times. Nevertheless, recent systematic reviews have highlighted that evidence regarding lateral thermal spread and tissue healing with these devices remains limited and inconsistent (24). In this regard, CUSA provides a distinct advantage by selectively fragmenting hydrated tissue with virtually no thermal propagation, thereby sparing adjacent reproductive structures—a feature particularly relevant in fertility-preserving endometriosis surgery.

CUSA has a relatively short and manageable learning curve, as demonstrated by Doron O. et al., who showed that both experienced surgeons and trainees achieved proficiency within a few attempts in a micro-neurosurgical model, aided by the device's intuitive handling and immediate feedback from its irrigation–aspiration system (25). This evidence aligns with our own operative experience and suggests that, although endometriosis surgery is technically challenging, CUSA is nonetheless applicable and can be integrated effectively into this surgical context. Its proven usability, together with its established role in other surgical specialties such as hepatobiliary surgery (26), supports its broader adoption in fertility-preserving endometriosis surgery. However, its implementation in routine gynecologic practice may be limited by availability in certain centers and by higher acquisition and maintenance costs compared with conventional energy devices, which could affect accessibility and cost-effectiveness, particularly in resource-constrained settings. In addition, the need for disposable handpieces further increases per-procedure costs, although operative times in our series were not prolonged, which may partly mitigate the economic impact. Beyond these economic aspects, wider adoption may also be constrained by limited availability across institutions, logistical barriers, and the requirement for structured training programs to ensure safe and consistent use in minimally invasive gynecology.

This study has some limitations that should be acknowledged. First, the small sample size and single-center setting may limit the generalizability of the findings. Additionally, as follow-up duration was relatively short, long-term outcomes related to symptom recurrence and fertility preservation could not be assessed. Finally, the subjective nature of the surgeon-reported scores could introduce observer bias, despite being performed by a highly experienced operator.

Future research should include multicenter studies with larger sample sizes to validate these findings and comparative trials assessing CUSA against other minimal thermal-spread technologies to establish its relative efficacy and safety. In addition to long-term symptom control and recurrence rates, future studies should explicitly incorporate reproductive endpoints, including postoperative conception rates and biomarkers of ovarian reserve, to clarify the impact of CUSA on fertility outcomes. A systematic evaluation of concomitant adenomyosis should also be considered, as it may influence symptom burden, surgical complexity, and postoperative recovery, and could modify the clinical outcomes observed in fertility-preserving surgery.

Exploring CUSA integration with robotic-assisted platforms and developing dedicated nerve-sparing protocols may further enhance its applicability in complex endometriosis cases. Combining robotic precision with CUSA's efficient tissue fragmentation and minimal thermal spread has shown promise in other complex surgeries, such as major hepatectomies, by reducing blood loss and improving dissection efficiency (27). However, challenges remain, particularly the need for robotic-specific CUSA tips and addressing the learning curve associated with device adaptation. Developing dedicated robotic-compatible instruments and conducting prospective studies could further optimize this approach and expand its clinical application.

This prospective case series demonstrates that the use of CUSA® for laparoscopic excision of endometriosis is both feasible and safe in the short term, achieving minimal blood loss, absence of intraoperative complications, and significant postoperative pain reduction. These findings highlight the technical precision and tissue-sparing potential of CUSA®, supporting its application in fertility-preserving surgery. Nevertheless, given the small sample size, single-center design, and lack of long-term and fertility-related outcomes, the results should be interpreted with caution. Further multicenter studies with larger cohorts and extended follow-up are warranted to validate these preliminary results and clarify the long-term clinical and reproductive benefits of this technique.

## Data Availability

The original contributions presented in the study are included in the article/Supplementary Material, further inquiries can be directed to the corresponding author.
