# Supplementary material for: Ultrasonic surgical aspiration (CUSA®) for laparoscopic excision of endometriosis: a prospective case series demonstrating safety and precision in fertility-preserving surgery
Source: Front Surg. 2025 Dec 16;12:1735940. doi: 10.3389/fsurg.2025.1735940 (PMC12752115; doi:10.3389/fsurg.2025.1735940)
Supplement: Supplementary file 1 [file Table1.docx]

Supplementary Material

# Supplementary Table

Supplementary Table 1 - Enzian classification by patient.

| Patient | #Enzian(s) |
| --- | --- |
| 1 | P2, B2/2 |
| 2 | P1, A1, B2/2 |
| 3 | B2/0, FD |
| 4 | A2, B2/2 |
| 5 | P2, T1/0, A1, B2/2 |
| 6 | T0/1, A3, B0/2 |
| 7 | P2, T1/0, B2/2 |
| 8 | A2, B1/2 |
| 9 | T1/0, B0/2 |
| 10 | T1/0, A2, B2/1 |
| 11 | T0/1, B0/1, FD |
| 12 | P1, T2/0, B2/1 |
| 13 | P2, O0/1, A2 |
| 14 | P2, O1/0, B3/1 |
| 15 | P2, A2, B1/1 |

#Enzian(s): surgical Enzian classification.
